# Fluorescence detection of white-beam X-ray absorption anisotropy: towards element-sensitive projections of local atomic structure

**DOI:** 10.1107/S0909049511030688

**Published:** 2011-09-15

**Authors:** P. Korecki, M. Tolkiehn, K. M. Dąbrowski, D. V. Novikov

**Affiliations:** a Institute of Physics, Jagiellonian University, Reymonta 4, 30-059 Kraków, Poland; b DESY, Notkestrasse 85, D-22603 Hamburg, Germany

**Keywords:** X-ray diffraction and absorption, atomic structure determination, polychromatic radiation, wavelet transform

## Abstract

Projections of the atomic structure around Nb atoms in a LiNbO_3_ single crystal were obtained from a white-beam X-ray absorption anisotropy (XAA) pattern detected using Nb *K* fluorescence. This kind of anisotropy results from the interference of X-rays inside a sample and, owing to the short coherence length of a white beam, is visible only at small angles around interatomic directions. Consequently, the main features of the recorded XAA corresponded to distorted real-space projections of dense-packed atomic planes and atomic rows. A quantitative analysis of XAA was carried out using a wavelet transform and allowed well resolved projections of Nb atoms to be obtained up to distances of 10 Å. The signal of nearest O atoms was detected indirectly by a comparison with model calculations. The measurement of white-beam XAA using characteristic radiation indicates the possibility of obtaining element-sensitive projections of the local atomic structure in more complex samples.

## Abstract 2

A method for a direct measurement of X-ray projections of the atomic structure is described. Projections of the atomic structure around Nb atoms in a LiNbO_3_ single crystal were obtained from a white-beam X-ray absorption anisotropy pattern detected using Nb *K* fluorescence.

## 1. Introduction

Direct visualization of atomic configurations around particular kinds of atoms using X-ray methods is cumbersome. While extended X-ray absorption fine structure (EXAFS) (Lee *et al.*, 1981 ▶) is a powerful technique, it provides information mainly about interatomic distances. In the last decade of the twentieth century, X-ray fluorescence holography (XFH) was introduced as a method which should be capable of providing three-dimensional images of the local structure around the chosen kinds of atoms (Tegze & Faigel, 1996 ▶; Faigel *et al.*, 2007 ▶). Though XFH is currently used for solving structural problems (Hosokawa *et al.*, 2009 ▶; Hu *et al.*, 2009 ▶; Happo *et al.*, 2011 ▶), the extinction phenomena, or, more generally, dynamical diffraction effects, can make the data interpretation difficult (Tegze *et al.*, 2002 ▶; Kopecky *et al.*, 2002 ▶; Korecki *et al.*, 2004*a* ▶).

In Korecki & Materlik (2001 ▶), a method for obtaining quasi-real-space projections of the averaged local atomic structure, by means of recording white-beam X-ray absorption anisotropy (XAA), was demonstrated. The basic idea of this approach is similar to XFH in a so-called reciprocal geometry (Gog *et al.*, 1996 ▶) or to the X-ray standing-wave (XSW) technique (Vartanyants & Kovalchuk, 2001 ▶). All these methods derive information from absorption effects (Cowley, 1964 ▶; Nishino & Materlik, 1999 ▶). They analyze the interference of the direct radiation with the radiation elastically scattered on atoms inside the sample. This interference modifies the total X-ray field at the sites of absorbing atoms. Therefore, the absorption cross section changes with the relative orientation of the sample and the incident-beam direction and can be written as 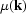 = 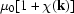, where 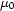 is the absorption cross section (per unit volume) calculated for isolated atoms and 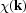 is the absorption anisotropy. In the experiments the absorption is probed by measuring the secondary yield 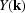, which is assumed to be proportional to absorption. Since characteristic radiation can be used for measuring 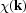, element-specific information is accessible. XFH and XSW data are recorded using monochromatic radiation and the structural information is sampled in reciprocal space. While XSW provides information about positions of absorbing atoms relative to a well ordered lattice, XFH is potentially suited for imaging of the local structure around absorbing atoms. In both cases, recorded data are converted to real space either by a holographic reconstruction (Barton, 1988 ▶) or by Fourier inversion procedures (Lee *et al.*, 2010 ▶).

**Figure 1:**
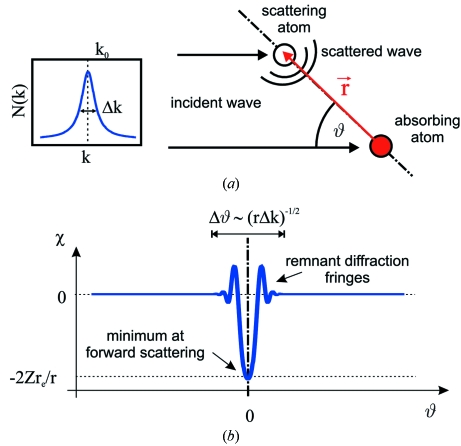
Origin and basic properties of white-beam XAA. (*a*) Two-atom system: absorbing and scattering atom. The total X-ray field amplitude at the site of an absorbing atom is the coherent sum of an incident plane wave and a scattered spherical wave. In an experiment the orientation of both atoms relative to the incident-beam direction is accomplished by the rotation of the sample. (*b*) Corresponding X-ray absorption anisotropy 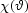. For white X-rays, owing to the short temporal coherence length, anisotropy is significant only at small angles around the interatomic direction. For 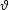 = 0, owing to a negative value of the X-ray scattering amplitude, there is always a minimum in the absorption anisotropy.

The situation changes when a broad-band polychromatic beam is used for recording of XAA. For a sufficiently broad-band spectrum, the coherence length of the radiation is so short that the interference of the direct and scattered radiation can only be observed near the forward scattering as demonstrated in Fig. 1 ▶. The effect of the localization of the signal in the vicinity of interatomic directions can be used to obtain quasi-real-space information about atomic structure. By changing the sample orientation one brings different inter­atomic directions into an orientation parallel to the beam direction, which results in a small decrease in X-ray absorption. For a crystal, one can thus observe the projections of dense-packed atomic planes and direction. By this means, though the principle is different, the XAA signal has some common features with medium and high electron diffraction/channeling patterns (Spence & Tafto, 1983 ▶; Osterwalder *et al.*, 1991 ▶; Day, 2008 ▶; Winkelmann *et al.*, 2008 ▶; Uesaka *et al.*, 2011 ▶). The signal is weak (the amplitude of white-beam XAA is of the order of 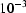) but it can be modeled using kinematical theory. For the same reason, in an appropriate experimental geometry, multiple-scattering effects can be eliminated (Korecki *et al.*, 2006*b* ▶).

A detailed description of white-beam XAA can be found by Korecki *et al.* (2006*b* ▶, 2009*a* ▶,*b* ▶). Here we only briefly describe the fundamental properties of this kind of anisotropy in X-ray absorption. The simplified form of the XAA signal can be written as
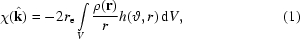
where 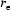 = 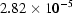
 Å is the Thomson scattering length, 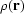 is the electron density of the sample occupying volume 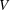, 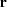 is the position relative to the absorbing atom and *r* = 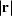. In accordance with the notation of Korecki *et al.* (2009*a* ▶) ▶, the unit vector 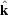 is antiparallel to the direction of the incident beam and 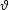 = 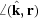. Assuming a Lorentzian energy spectrum of the incident beam, which is centered at the wavevector value 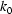 and having a full width at half-maximum (FWHM) equal to 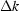, 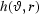 can be written as
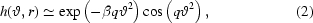
where *q* = 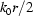 and β = 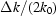. At the exact forward-scattering condition the anisotropy can be approximated as χ(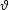 = 0, *r*) ≃ 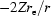, where 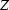 is the atomic number of the scattering atom. In (2) a small-angle approximation was used. It is justified only for a broad-band spectrum with a short coherence length. Since the scattering is limited to small angles, the polarization factor can be neglected without significant loss of accuracy.

XAA data can be analyzed using different approaches. First, the data can be directly compared with geometrical projections of the structure (Korecki *et al.*, 2009*b* ▶). This analysis is only qualitative since the remnant diffraction slightly distorts the projections of atomic planes and directions. Second, a tomographic algorithm can be used to visualize the positions of the absorbing atoms relative to a periodic lattice (Korecki *et al.*, 2006*c* ▶). It is based on a quantitative analysis of the intensity and shape of bands corresponding to real-space projection of atomic planes. This approach is so far limited to cubic samples and will be not discussed here. Third, a spherical wavelet transform can be used to suppress the contributions from distant atoms, providing the projections of local atomic structure (Korecki *et al.*, 2009*a* ▶).

In all previous attempts, white-beam XAA was recorded using the total electron yield. While the total electron yield can be measured very efficiently, it allows for element sensitivity only in special cases, when absorption on particular atoms is dominant.

In this work we measured white-beam XAA using characteristic radiation, *i.e.* X-ray fluorescence. Although the measurement was performed for LiNbO_3_, where only Nb atoms emit fluorescence in the hard X-ray range, the present work describes a necessary step towards application of white-beam XAA for analysis of real structural problems.

The paper is organized as follows. In §2 we describe details of the experimental set-up, data acquisition and data processing. In §3 we present experimental XAA patterns and discuss them by comparison with simulations. The quantitative data analysis is performed using the wavelet transform. The final section is devoted to conclusions.

## 2. Experiment and data processing

### 2.1. Sample

As a sample we used a *Z*-cut (001) LiNbO_3_ single crystal with a diameter of 10 mm. For a review of its structural and physical properties including references, see Weis & Gaylord (1985 ▶).

**Figure 2:**
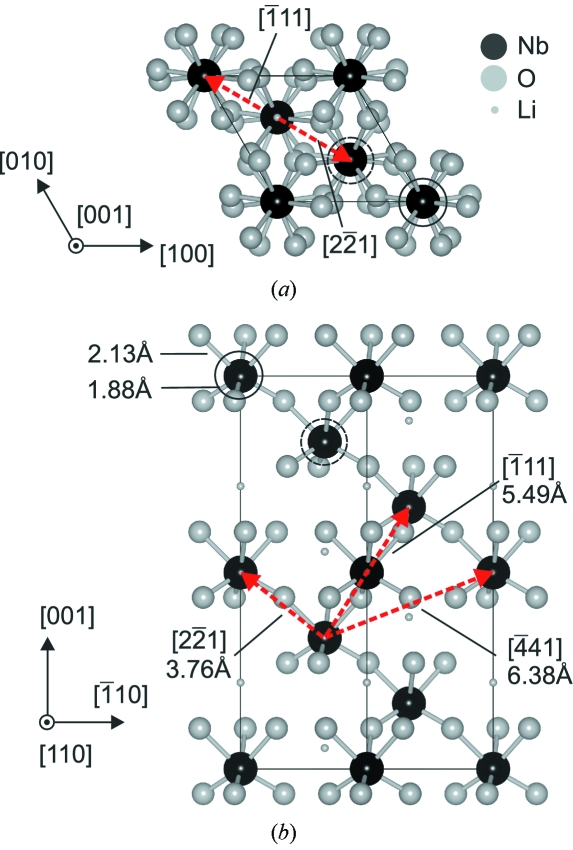
Structure of LiNbO_3_. (*a*) and (*b*) show views along the [001] and [110] directions, respectively. The hexagonal unit cell is shown using solid lines. Dashed arrows depict shortest Nb—Nb bonds. Solid and dashed circles mark two non-equivalent positions of Nb atoms, for which the oxygen octahedra have different orientations. They are mirrored with respect to the (110) plane.

The room-temperature structure of the ferroelectric LiNbO_3_ (space group 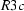, hexagonal unit cell with *a* = *b* = 5.148 Å, *c* = 13.863 Å, α = β = 90°, γ = 120°) can be seen as hexagonal closed-packed planar sheets of O atoms (Abrahams *et al.*, 1966 ▶). This structure is visualized in Fig. 2 ▶ using *VESTA* software (Momma & Izumi, 2008 ▶). The octahedral interstitials are filled with Nb, Li or a vacancy. In the unit cell there are six Nb, six Li and 18 O atoms. The Nb and Li atoms are shifted from the symmetrical positions relative to the oxygen planes along the [001] direction.

LiNbO_3_ was used in this feasibility study for the following reasons. First, hard X-ray fluorescence is emitted only by Nb atoms. This allows us to use a detector with a moderate energy resolution. Second, it will be possible to demonstrate that the wavelet transform can be successfully applied to the XAA data recorded on a limited angular range, for a sample having a lower symmetry compared with samples usually examined by XFH. In XFH the missing data problem can strongly influence the data quality. Therefore, holograms are frequently extended to a full sphere using a symmetrization procedure (Tegze *et al.*, 1999 ▶). Otherwise, the quality of the real-space reconstruction can be degraded (Marchesini *et al.*, 2000 ▶; Hayashi *et al.*, 2007 ▶). Furthermore, there is a large difference between the atomic numbers of niobium and oxygen. In addition, O atoms have a relative 0.5 occupancy in the averaged local structure of Nb atoms. This will allow us to check whether the weak signal from O atoms can be detected despite a strong contribution from Nb.

### 2.2. Experimental set-up

**Figure 3:**
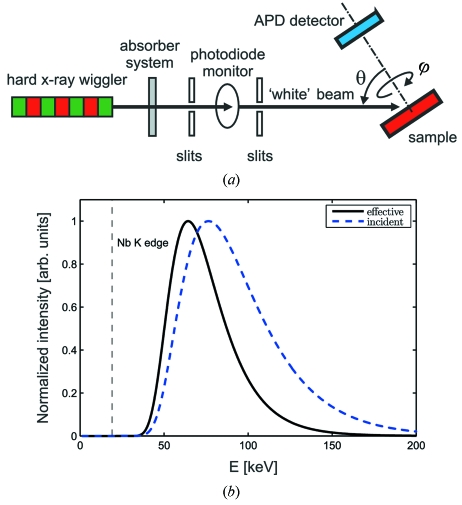
(*a*) Experimental set-up for the measurement of white-beam XAA. Radiation from a hard X-ray wiggler was passed through a set of absorbers in order to produce a broad-band energy spectrum and to shift it towards higher energies. The beam intensity was monitored using a photodiode. XAA was obtained from a two-dimensional dependence of the absorption measured while the sample was rotated relative to the incident-beam direction around two axes. The Nb *K* X-ray fluorescence, which was used to probe the absorption at atomic sites, was measured using an avalanche photodiode. (*b*) Calculated energy spectra; number of photons per second per energy interval. Dashed line: energy spectrum of the incident beam. Solid line: effective energy spectrum, which includes absorption dependence inside the sample. Both curves were normalized to their maxima. The vertical line depicts the position of the Nb *K* absorption edge.

The experiment was carried out at the hard X-ray wiggler beamline BW5 at the DORIS III storage ring at DESY (Bouchard *et al.*, 1998 ▶). The scheme of the experimental set-up is shown in Fig. 3(*a*) ▶. The direct beam from the wiggler passed through a set of absorbers (1.5 mm-thick Cu, 4 mm Al, 4 mm glassy carbon) which acted as a high-pass energy filter and allowed the spectrum of the radiation to be hardened. The beam was geometrically shaped using systems of slits to a square shape with dimensions 1 mm × 1 mm. The beam intensity was monitored using a Si X-ray photodiode placed directly in the beam path.

The calculated energy spectrum of the incident radiation and the effective energy spectrum sensed by Nb atoms are shown in Fig. 3(*b*) ▶. The sensed radiation spectrum is calculated from the incident spectrum by multiplying it by the energy dependence of the X-ray absorption inside the sample (Korecki *et al.*, 2006*b* ▶). For the purpose of data evaluation, the effective spectrum is most important: it determines all properties of XAA. Both spectra were calculated using procedures from the X-ray optics package *XOP2.3*. The big advantage of white-beam XAA is that it is robust to relatively large variation of the spectrum shape (Korecki *et al.*, 2006*a* ▶). Thus, the spectrum does not need to be precisely measured during the experiment. The effective energy spectrum has a maximum at 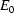 ≃ 64 keV (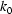 ≃ 33 Å^−1^) and FWHM 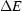 ≃ 36 keV (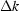 ≃ 18 Å^−1^). For such a beam the longitudinal coherence length is below 0.35 Å. As compared with the incident-beam spectrum, the effective spectrum is shifted towards lower energies. However, the lower-energy cut-off is well above the Nb *K*-edge ensuring a smooth spectrum without any jumps corresponding to absorption edges. For grazing angles the absorption length of the incident beam and the escape depth of the Nb *K* fluorescence become comparable and the effective radiation spectrum shifts towards higher energies owing to the beam-hardening effect.

The white-beam XAA was obtained from a two-dimensional dependence of the Nb *K* fluorescence yield measured while the sample was rotated relative to the incident-beam direction around two axes. For the measurement we used a specialized apparatus, which was originally designed for measurement of kinematical X-ray standing waves and multiple-energy X-ray holography (Tolkiehn *et al.*, 2005 ▶). The spectrometer combined a standard stepper-motor-driven rotation stage with a fast servo-motor-driven rotation axis. During the experiment both the fast axis (rotation around sample normal, ϕ) and slow axis (deviation from the normal to the sample surface, θ) rotated continuously at a constant speed and were synchronized. This corresponded to a spiral acquisition scheme.

The Nb *K* X-ray fluorescence was measured using an avalanche photodiode (APD), which rotated together with the slow motor θ. The APD (area of 5 mm × 5 mm and thickness of 110 µm) was mounted on the ϕ axis at a distance of 80 mm above the sample surface. Thus, the ϕ motion did not change the relative orientation of the sample and APD. While an APD is quite efficient for detection of Nb *K* fluorescent photons (∼20%) it has a very low efficiency for higher-energy photons (<1% at 50 keV). Though the APD has sufficient resolution to distinguish between the Nb *K* fluorescence photons and the elastic and Compton ones, only a lower level discriminator was used. As checked before the experiment, the higher-energy photons did not produce a significant signal in the detector. This was also confirmed by the lack of Bragg spots in the measured XAA. Simultaneously, if pile-up is allowed during the detection, the APD can achieve larger counting rates (Walko *et al.*, 2008 ▶), which cannot, however, exceed the bunch frequency (5.2 MHz for Doris III).

During the rotation, data were continuously acquired over 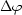 = 0.2° intervals, which corresponded to an acquisition time per point of ∼0.05 s and to 1800 points per one spiral revolution. The slow angle θ was changed from 33° to 89° with the spiral jump 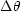 = 0.5°. A single data set with over 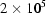 points was measured in ∼3 h. In total, seven similar runs were performed. The count rate of the APD changed from 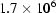
 Hz for 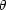 ≃ 33° to 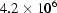
 Hz for 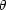 ≃ 89°.

### 2.3. Data processing

X-ray absorption anisotropy was obtained from the two-dimensional dependence of the fluorescence yield. Prior to the background subtraction the raw data were dead-time corrected and normalized by the monitor signal. The correction of the non-linearity of the APD detector, resulting from dead-time effects, was performed based on a so-called ‘isolated’ model characteristic of a pulsed synchrotron source and counting using a lower-level discriminator (Walko *et al.*, 2008 ▶). The XAA pattern 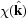 was computed accordingly to 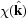 = 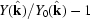, where 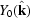 is the slowly varying background. The exact form of the slowly varying background is difficult to model for a white-beam illumination; it requires the exact knowledge of the spectrum. For data evaluation purposes, less cumbersome procedures are sufficient. Therefore 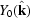 was determined using smoothing splines in *MATLAB Splines Toolbox*. Splines are functions defined piecewise by polynomials. By using a single parameter of a smoothing spline, which controls the trade-off between goodness-of-fit to the data and roughness of the splines, one can determine the shape of the background. For extreme values of this parameter one can produce a least-squares straight-line fit to the data or a cubic spline, *i.e.* a smooth curve joining all the data points. The optimal value of this parameter was found using a trial-and-error procedure.

After background subtraction the data were symmetrized using a threefold symmetry axis and a mirror plane. Because of the high noise level introduced by a strong non-linear dead-time correction at counting rates approaching bunch frequency, the data range was restricted to angles 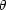 < 83°. All XAA patterns are presented as stereographic projections, *i.e.* the coordinates 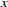 and 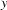 in the images are related to angles in spherical coordinates by *x* = 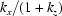, *y* = 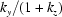, where 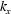 = 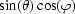, 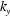 = 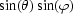 and 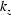 = 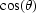.

The wavelet transform applied in the following section to the XAA data was performed in *MATLAB* using spherical continuous wavelet transform procedures from the *YAWTb* toolbox (Jacques *et al.*, 2001 ▶).

## 3. Analysis of X-ray absorption anisotropy

### 3.1. Experimental absorption anisotropy

**Figure 4:**
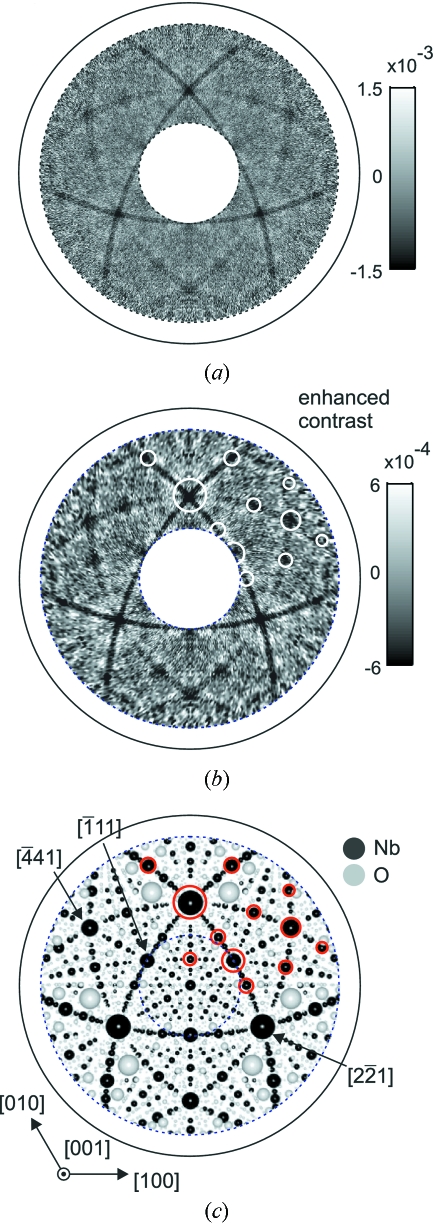
White-beam XAA recorded for a LiNbO_3_ crystal using Nb *K* fluorescence. Panels (*a*) and (*b*) show the same data. In (*b*) the contrast was improved for better visibility. The patterns are presented as stereographic projections. (*c*) Stereographic view of the model of the local structure around Nb atoms calculated for a cluster with a radius of 25 Å. Spheres corresponding to O atoms were enlarged by a factor of four.

Fig. 4 ▶ shows a white-beam XAA pattern recorded for the LiNbO_3_ sample using a Nb *K* fluorescence signal. For a better visibility the data presented in Fig. 4(*b*) ▶ are shown with a highly enhanced contrast as compared with the full-range intensity scale of Fig. 4(*a*) ▶. The outer solid circle corresponds to 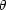 = 90°.

Fig. 4(*c*) ▶ shows a stereographic model view of the averaged local structure around Nb atoms calculated for a cluster of radius 25 Å using the *POVRAY* ray-tracer. The observation point is located at a Nb site. The radius of spheres is proportional to the atomic number, effective occupancy and is inversely proportional to the radial distance of a given atom from the central Nb atom. For a better visibility the spheres corresponding to O atoms are enlarged by a factor of four. The Li atoms are not shown.

As seen from the comparison of Figs. 4(*b*) and 4(*c*) ▶, the most apparent features of the anisotropy are dark bands corresponding to the projections (so-called traces) of dense packed Nb planes. The dark spots correspond to zone axes, *i.e.* to the dense packed atomic rows in the crystal. Features corresponding to O atoms cannot be directly observed in the measured patterns.

Despite the small value of the anisotropy, the measured XAA pattern provides direct real-space information about the atomic structure of the sample. This kind of information is unique among X-ray methods.

### 3.2. Calculated absorption anisotropy

Prior to the extraction of more quantitative information from the XAA pattern, it is necessary to show that it results from single-scattering processes, *i.e.* it can be modeled using kinematical theory. The formalism needed for a calculation of XAA was presented by Korecki *et al.* (2006*b*,  ▶2009*a* ▶). In brief, the XAA pattern can be calculated using two approaches. First, the X-ray field at the sites of absorbing atoms can be calculated by summing the contributions from individual atoms using equation (1). Second, the electron density inside a crystal can be represented as a Fourier series. Then, the calculation can be performed as a summation in reciprocal space (Adams *et al.*, 1998 ▶; Marchesini *et al.*, 2002 ▶). In principle, both approaches are equivalent, *i.e.* the summation over an infinite number of terms (atoms or reciprocal space vectors) should give the same result (Winkelmann *et al.*, 2008 ▶). In practice, both approaches are low convergent and a kind of Ewald summation would be very useful (Ewald, 1921 ▶).

**Figure 5:**
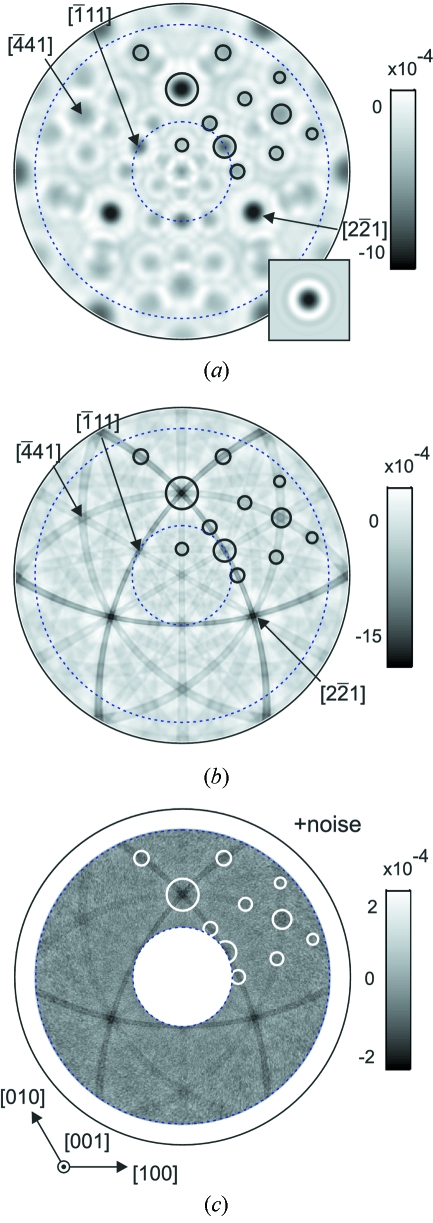
White-beam XAA calculated for a LiNbO_3_ crystal. (*a*) XAA calculated by a direct real-space summation inside a cluster with a radius of 12 Å. The inset shows a signal from a single scatterer located at a distance of 3.76 Å (shortest Nb—Nb distance). (*b*) XAA pattern calculated using a reciprocal summation. (*c*) Same as (*b*) but the noise was added and the angular range was limited for a direct comparison with the experimental pattern from Fig. 4 ▶.

In the calculation the effective radiation spectrum had a Lorentzian shape with a maximum at 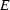 = 64 keV and a width of 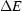 = 36 keV. In the unit cell of LiNbO_3_, Nb atoms occupy non-equivalent positions relative to the oxygen sublattice. Therefore the calculated XAA presented in Fig. 5 ▶ is an average of XAA characteristic of different absorbing sites of Nb. The calculated data are presented using the same stereographic grid as the experimental data.

The XAA shown in Fig. 5(*a*) ▶ was calculated using a direct summation over atoms in a cluster with a radius of 12 Å around the Nb absorbing atoms. The inset (bottom right) shows the XAA signal corresponding to a single Nb atom placed at the shortest Nb—Nb distance. For such a small cluster, signals from all Nb atoms can be directly recognized in the data. Owing to a high angular resolution, resulting from the use of a hard X-ray wiggler, the individual signals are well separated, *i.e.* there is only a minor overlap between them. The signals coming from O atoms are hardly visible in the data owing to the difference in atomic numbers of Nb and O and a relative 0.5 occupancy of O atoms in the averaged local structure of Nb.

As seen both in the inset and in the main figure, all atomic signals consist of a distinct zero-order minimum corresponding to the forward-scattering direction and strongly suppressed higher-order interference rings. Note that features which correspond to the first-order maxima (white rings) can also be recognized around dark minima in the experimental XAA from Fig. 4 ▶.

The XAA shown in Fig. 5(*b*) ▶ was calculated using reciprocal-space summation (Korecki *et al.*, 2006*b* ▶). In this pattern the bands corresponding to the projection of atomic planes are clearly visible. In order to facilitate a direct comparison of the calculated and measured data, Fig. 5(*c*) ▶ presents the calculated data, restricted to the same angular range as in the experiment. In addition, Poisson noise at the level corresponding to the experimental situation was added to the data. The agreement between calculation and experiment is excellent, proving the lack of extinction and/or multiple-scattering effects.

### 3.3. Wavelet transform of absorption anisotropy

In a recent work (Korecki *et al.*, 2009*a* ▶) a continuous spherical wavelet transform (Antoine *et al.*, 2002 ▶) was used for a quantitative analysis of XAA patterns. Continuous wavelet transform (Farge, 1992 ▶) is frequently applied for analysis and feature detection in signals and images. Continuous wavelet transform is often compared with the Fourier transform. While Fourier transform is limited to frequency analysis, wavelets can be utilized for both frequency and spatial analysis. Therefore, wavelets can be used for the detection of localized features. Wavelets have found applications in various physical disciplines (van den Berg, 2004 ▶) including optics. For example, wavelet transform was used for description of diffraction phenomena (Onural, 1993 ▶) and for reconstruction of visible-light holograms (Buraga-Lefebvre *et al.*, 2000 ▶).

A family of wavelets can be constructed from a single function, a so-called ‘mother wavelet’, which meets a so-called admissibility condition; roughly speaking it has a zero mean. All other wavelets are then formed by translation (or rotation in the case of sphere) and scaling and have the same universal shape. A particular form of the mother wavelet can be adopted for a specific application. In most applications wavelets are well localized oscillatory functions. The continuous wavelet transform corresponds to a decomposition of the analyzed signal into wavelets, which can be described as a generalized correlation between the signal and the scaled and translated wavelets.

Korecki *et al.* (2009*a* ▶) ▶ showed that, if a white beam is used for recording of XAA, the signals from individual atoms have the same universal shape and differ only in the scale and angular position. Thus, the XAA pattern can be described as a simple linear superposition of wavelet-like functions, each corresponding to a single scatterer. Therefore, the wavelet transform is a natural and perhaps an optimal method of analyzing the XAA patterns recorded for white X-rays and imaging of the local atomic structure.

For this purpose we used an isotropic wavelet family defined as
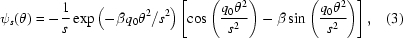
where θ is a polar angle, 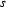 is a scale parameter and 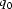 = 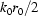. The parameter β has already been used in equation (2) and describes the energy spectrum, and 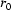 is another parameter set to a minimal possible value which ensures the validity of the small-angle approximation for scale parameters 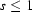.

The presence of the cosine term in (3) ensures that wavelet 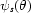 is very well matched to the signal 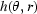 from (2) for a scale parameter
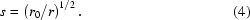
The much smaller sine term is a correction introduced to meet the zero mean condition.

For isotropic wavelets the continuous spherical wavelet transform can be defined as a generalized spherical correlation between scaled and rotated wavelets and the XAA signal according to (Antoine *et al.*, 2002 ▶)
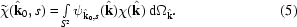
The subscript 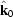 in 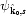 denotes that the wavelet 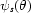 from (3) was rotated from the north pole to the direction determined by 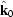.

Note that the scaling operation of wavelets defined by (3) is a simplified version of this procedure performed in the genuine spherical wavelet transform. The spherical wavelet transform is locally equivalent to a two-dimensional wavelet transform (Wiaux *et al.*, 2005 ▶), and (3) is valid only under the small-angle approximation, *i.e.* when wavelets are well localized and the curvature of the sphere can be neglected. As seen from (3) and (4), the localization of the wavelets is solely determined by 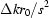 = 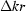. Thus, the broader the incident-beam spectrum the shorter the interatomic distance 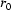 at which the small-angle approximation holds and the simplified version of the spherical wavelet transform can be used.

Since the wavelets from (3) are well matched to the signals of individual scatterers, the coefficients of the wavelet transform will be at a maximum if the scale and position of the wavelet coincide with the distance and direction between scattering and absorbing atoms, respectively. This effect is directly connected with the correlation-like character of the wavelet transform. The intensity of the maxima will be inversely proportional to the distance between the absorbing and scattering atoms and proportional to the ratio of the atomic number and the effective occupancy of the scatterer.

A more quantitative interpretation of the wavelet transform coefficients can be established based on the comparison of white-beam XAA with XFH. In XFH the reconstruction of a hologram provides a distorted image of the electron density. The reconstructed electron charge distribution is convolved with an experimental point-spread function (Marchesini & Fadley, 2003 ▶; Saldin *et al.*, 1991 ▶). The exact shape of the point-spread function depends on the beam energy and on the angular range of the data. The wavelet transform can be described as a holographic reconstruction (with a slightly modified ‘admissible’ kernel), in which the XAA data are additionally multiplied by a window function that is rotated on the sphere and scaled depending on the real-space distance. Thus, the wavelet transform coefficients also correspond to the convolution of the averaged electron density distribution with a point-spread function, which for a white X-ray beam becomes highly elongated in the radial direction. Note that, because of the localization of the white-beam XAA around interatomic directions, the direct application of holographic reconstruction to white-beam XAA data will not improve the accuracy, but it will induce massive artefacts to the real-space images. Also note that the application of wavelet transform to XAA data does not produces twin images (Len *et al.*, 1994 ▶).

**Figure 6:**
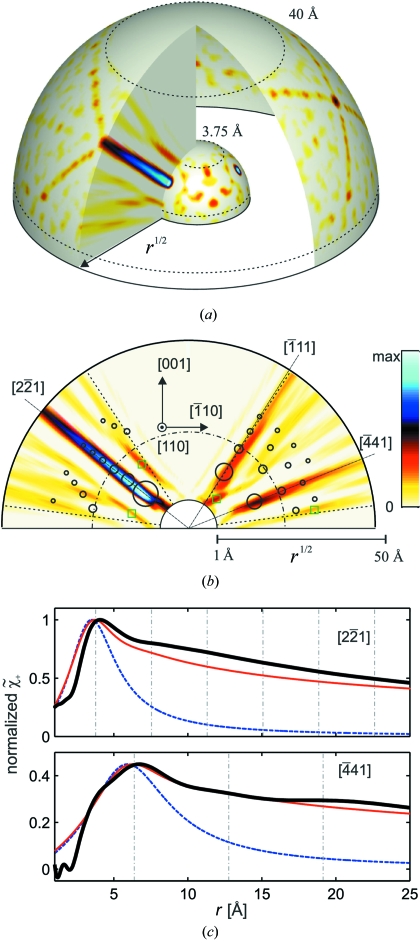
Wavelet transform of the experimental XAA pattern. (*a*) Three-dimensional view of the wavelet transform coefficients 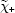 showing two spherical shells at distances of 3.75 Å and 40 Å and a single radial slice. (*b*) A radial slice of wavelet transform 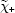. The dashed circles and lines correspond to the angular range where the data were recorded, small circles mark the positions of Nb atoms, and squares mark artefacts. A false-color scale was used in order to show both strong and weak features. For a better visualization the radial coordinate is equal to the square root of the real-space distance. The dash-dotted hemicircle has a radius of 12 Å. (*c*) Profiles of 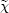 along the 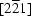 and 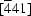 directions. The thick solid (black) lines show the experimental wavelet coefficients. The dashed (blue) lines present the theoretical wavelet transforms calculated for nearest Nb atoms located along corresponding directions. The thin solid (red) lines correspond to the theoretical wavelet transforms calculated for single periodic atomic rows. The dash-dotted lines mark the position of Nb atoms in these rows.

The continuous spherical wavelet transform maps two-dimensional XAA data sampled on a spherical surface onto a three-dimensional spherical volume as demonstrated in Fig. 6(*a*) ▶. The wavelet transform coefficients shown here were computed from the experimental XAA pattern. Fig. 6(*a*) ▶ shows two spherical shells calculated for constant distances 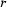 = 3.75 Å and 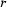 = 40 Å and a single radial slice. The angular position on the sphere corresponds to the position of the wavelet on the sphere, whereas the radial coordinate corresponds to the square root of the real-space distance and not, as usual, to the scale of wavelets. For presentation purposes it is convenient to show the wavelet coefficients using such a radial scale and a modified function 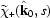, that is equal to 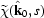 for positive values and zero otherwise. However, in order to show both the high- and low-valued positive coefficients, a false-color scale is used for presentation.

For the purpose of a quantitative analysis, Fig. 6(*b*) ▶ shows a (110) radial slice in a conventional two-dimensional presentation (*c.f.* Fig. 2*b* ▶). The radial coordinate starts from *r* = 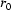 = 1 Å and ends at ∼50 Å. For distances smaller than 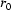 = 1 Å a simple scaling operation of the wavelets is not valid and the wavelet transform was not computed in this range. The solid circles mark the positions of Nb atoms. Their dimensions are inversely proportional to the distances from the absorbing site. Fig. 6(*c*) ▶ presents radial profiles of the wavelet transform along the 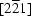 and 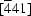 directions, *i.e.* passing through the most intense spots. Theoretical wavelet transforms of calculated XAA taking into account either only the nearest Nb atom located along corresponding directions or a single periodic atomic row are shown for comparison. Even such a simplified calculation reproduces the character of the experimental results.

The most apparent observation is that all shapes in the plots are strongly elongated. This is a direct consequence of the deterioration of the radial resolution resulting from the low coherence length of the radiation. The radial resolution, which degrades with 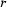, is given by 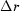 = 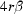 (in this work 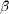 ≃ 0.3). The most intense elongated spots are located at directions coinciding with the shortest Nb—Nb distances. Simultaneously, these directions have the most dense atomic packing and the elongation of spots is enhanced by the presence of atoms located at larger distances in the same atomic row. The two strongest spots have maxima at 4.03 ± 0.12 Å and 6.75 ± 0.25 Å. The corresponding Nb—Nb distances are 3.76 Å and 6.38 Å, respectively. The discrepancy in these values is caused mainly by the approximation of the incident-beam spectrum with a Lorentzian curve. A secondary effect causing the shift of the maxima is connected with the visible overlap with the images from atoms at larger distances, as shown in Fig. 6(*c*) ▶. Individual atoms in 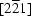 and 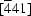 atomic rows cannot be resolved owing to poor radial resolution. Consequently, one is only able to determine the shortest bond lengths along a given direction with an accuracy of the order of 0.5 Å.

There are two kinds of artefacts visible in the radial cut. The first kind of artefact is connected with the non-othogonality of wavelets, which effects in spurious oscillations in the transform. Such oscillations can also be observed in single-energy X-ray and γ-ray holography and can be modeled using a so-called point-spread function (Marchesini & Fadley, 2003 ▶; Korecki *et al.*, 1997 ▶, 2004*b* ▶). However, one can observe that artefacts and the true atomic images show a very distinct behavior. All atomic spots are elongated exactly along the radial directions. Any other spots visible in the pattern, which have a non-radial elongation, can be classified as artefacts. Several such spots are visible in Fig. 6(*b*) ▶ and are marked with squares. For a better inspection of this effect, auxiliary radial lines are plotted. The second kind of artefacts results from edge effects. Images of all atoms lying in the vicinity of edges are distorted (*e.g.* the atom located in the 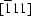 direction). In the wavelet terminology the edges lie in their cones of influence. Such artefacts also show a non-radial behavior.

There exists a possibility to reduce artefacts at the expense of the loss of radial resolution. The wavelet transform is invertible; it is guaranteed by the admissibility condition. This means that the original signal can be reconstructed from a full set of wavelet coefficients (Farge, 1992 ▶). Consider a linear inversion scheme of the wavelet transform, in which one takes wavelet coefficients corresponding to a limited range of scales or distances starting from *r* = 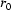 and ending at the cut-off distance *r* = 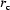. Such a modified inversion can be interpreted as a wavelet filter which suppresses fine details from the pattern, *i.e.* it reduces contribution from scatterers, which are far away. More precisely the images of atoms located at *r* < 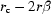 will be relatively weakly influenced by the filter, whereas the images of more distant atoms will be strongly suppressed. This kind of filter has quite a fuzzy cut-off resulting from a poor radial resolution. After application of the wavelet filter, because of the radial averaging, the images of near atoms will be enhanced relative to artefacts. This is due to the different behavior of the true images and artefacts along the radial direction.

**Figure 7:**
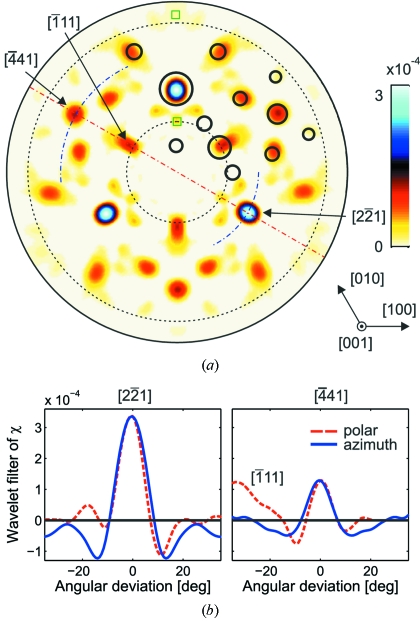
X-ray projection of the local structure around Nb atoms; wavelet filter calculated for a cut-off value of 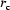 = 12 Å. (*a*) Stereographic view. Negative values were put to zero. Both the angular and intensity scales can be directly compared with images from Fig. 5 ▶. The dashed circles correspond to the angular range where the data were recorded, small circles mark the positions of Nb atoms, and squares marks artefacts. (*b*) Angular profiles taken along the dash-dotted lines shown in (*a*). Solid and dashed curves show azimuthal and polar profiles, respectively.

A wavelet filter of the experimental XAA pattern calculated for 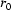 = 1 Å and 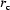 = 12 Å is shown in Fig. 7 ▶. It can be interpreted as a quasi-real-space projection of the local structure around Nb atoms. Images of Nb atoms up to a distance of ∼10 Å can be identified in the pattern. The artefacts are strongly suppressed. The most intense spots, corresponding to atoms in the 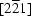 and 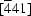 directions, have widths (defined as the angular distance between the zeros) of less than 18° and 14.5°, respectively. The accuracy in the determination of the maxima is limited by the possible overlap with other peaks, angular resolution and the uncertainty of sample orientation. In this work an accuracy of better than 0.5° was achieved for both azimuthal and polar directions. This number corresponds to the maximal observed difference between the determined and the theoretical values. The angular accuracy in the determination of atomic positions can be converted to the distances in the tangential planes, which are equal to 0.04 Å and 0.06 Å for the nearest atoms located in the 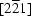 and 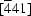 directions, respectively.

Until now the analysis was restricted to the identification of structural features coming from scattering on heavy Nb atoms. While the shortest Nb—O bond length (1.89 Å) is shorter than the Nb—Nb bond length (3.76 Å), there is a large difference in the atomic numbers of oxygen and niobium and in the effective occupancy.

**Figure 8:**
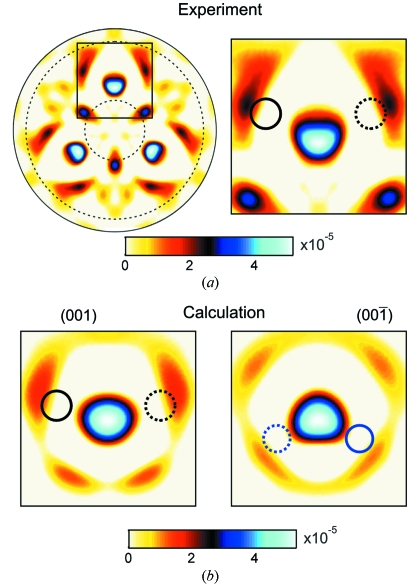
Indirect detection of the signal corresponding to O atoms. (*a*) Wavelet filter of experimental XAA pattern computed for a very small cut-off 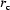 = 2 Å. The right-hand image shows an enlarged region depicted with a square. (*b*) Wavelet filters computed from simulated XAA patterns. Left- and right-hand images correspond to opposite sample orientations, for which Nb atoms are located at different distances below the oxygen planes along the [001] direction. The shape of the Nb atom image (strongest maximum) is distorted owing to the presence of O atoms marked with circles. The solid and dashed circles reflect positions of the O atoms from differently oriented octahedra (*c.f.* Fig. 2 ▶).

Consequently, the images of O atoms are not directly visible in the wavelet transform from Fig. 7 ▶. Korecki *et al.* (2009*a*) ▶ demonstrated that while the features resulting from scattering on lighter atoms are not always directly resolved in a wavelet transform of the XAA pattern, they can distort the shape of peaks resulting from scattering on heavy atoms, making their shape non-circular. It results from the straightforward real-space interpretation of white-beam XAA. In Fig. 8 ▶, a wavelet filter of XAA was calculated for a very small value of the cut-off distance 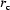 = 2 Å. Similarly, as in Fig. 7 ▶, the most intense spots correspond to the Nb atoms. Now, these images have a distorted triangular shape. Taking into account the position of O atoms (marked by circles) it can be concluded that this distortion is due to the signal of O atoms. This observation is confirmed by the analysis of wavelet filters computed from simulated XAA images. The simulated XAA patterns (not shown here) were calculated for two opposite sample orientations, for which Nb atoms are located at different distances below the oxygen planes along the [001] direction (*c.f.* Fig. 2 ▶). The symmetry of the Nb peak for the (001) orientation is the same as in Fig. 7(*a*) ▶. Since the calculation included only the scattering on Nb and O atoms located at distances less than the next-nearest Nb—Nb distance, it can be concluded that the change of the peak shape is solely due to overlap with signals of atoms in the local structure. For such a small value of 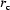, the artefacts resulting from non-orthogonality of the wavelets are not removed from the data. Thus, both in the experimental and simulated data one can observe a strong ring-like feature around the image of the Nb atom coming from higher-order interference fringes. Note, however, that the intensity of this ring is enhanced near the position of the O atoms in the same way in the experiment and simulation. Taking into account the very small signal (<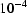), the agreement between the experimental and simulated images is very good. The main differences are due to the imperfect background subtraction. For near atoms, which produce signals corresponding to large scales, the background subtraction using splines can influence the atomic images. Note that the imaging of O atoms was demonstrated with XFH only once for a much simpler NiO structure with significantly smaller difference of atomic factors of Ni and O (Tegze *et al.*, 2000 ▶).

## 4. Conclusions

In this paper we showed that white-beam XAA can be measured using X-ray fluorescence for probing the X-ray field at the atomic sites. This indicates that the use of fast energy-resolving detectors would give the possibility of quasi-real-space element-sensitive imaging of the local atomic structure in more complex systems. For this purpose, multi-element silicon-drift detectors could be applied (Welter *et al.*, 2009 ▶).

The white-beam XAA has a small amplitude. However, simultaneously, the weakness of the signal allows for a straightforward interpretation of the data. In addition, as demonstrated in this work, XAA can be a photon-in/photon-out technique which will allow for studies in high electric or magnetic fields.

The application of wavelets allowed for a quantitative analysis of XAA patterns for a high-resolution determination of interatomic directions, *i.e.* for obtaining projections of local atomic structure and for a low-resolution determination of atomic bonds, which can help to interpret the projected data. A simple method for identifying and reducing artefacts in XAA was proposed. While images of strong Nb scatterers were imaged with high quality, the images of weak O scatterers were not directly visible. The determination of their position required comparison with simulated images. Thus, the imaging of systems in which there is a big difference in atomic numbers of the elements will be cumbersome but most probably possible. It is worthwhile checking whether an extension of the tomographic approach (Korecki *et al.*, 2006*b* ▶) to non-cubic samples could permit determination of the positions of Nb atoms relative to the oxygen planes.

White-beam XAA will benefit from using even harder X-rays than those produced by the hard X-ray wiggler at the DORIS ring. For example, the intense hard X-ray radiation from damping wiggler beamlines, planned in the PETRA III extension, would be ideally suited to performing white-beam XAA experiments.
